# Clinical and Functional Heterogeneity of COPD Phenotypes: A Multicenter Study from Turkey (DIPTUR Study)

**DOI:** 10.3390/medicina62020402

**Published:** 2026-02-19

**Authors:** Tevfik Ozlu, Ozlem Sengoren Dikis, Fulden Cantas Turkis, Ceren Degirmenci, Ahmet Ilgazlı, Inci Gülmez, Burcu Yalcin, Gulistan Karadeniz, Yasemin Soyler, Hatice Selimoglu Sen, Aysel Sunnetcioglu, Nimet Aksel, Sibel Boga, Nurhan Sarioglu, Haci Ahmet Bircan, Aylin Capraz, Serap Argun Baris, Aycan Yuksel, Umut Sabri Kasapoglu, Sibel Arınc, Esra Yarar, Nur Aleyna Yetkin, Fusun Sahin, Ali Tabaru, Dildar Duman, Gunhan Yavasoglu, Dursun Tatar, Mehmet Karadag, Kadir Coban, Ersin Alkilinc, Ebru Tas, Taha Tahir Bekci, Derya Kizilgoz, Buket Mermit, Murat Kavas, Hakan Alp Yilmazli, Ilknur Basyigit, Esen Sayin Gulensoy, Meltem Agca, Filiz Alkan Baylan, Canan Bol, Berat Uslu, Gamze Celik

**Affiliations:** 1 Department of Pulmonary Medicine, Faculty of Medicine, Karadeniz Technical University, Trabzon 61080, Turkey; ozlutevfik@yahoo.com (T.O.); kadircobanmd@gmail.com (K.C.); 2 Department of Pulmonary Medicine, Faculty of Medicine, Mugla Sitki Kocman University, Mugla 48000, Turkey; 3 Department of Biostatistic, Faculty of Medicine, Mugla Sitki Kocman University, Mugla 48000, Turkey; 4 Department of Pulmonary Medicine, Muğla Training and Research Hospital, Mugla 48000, Turkey; cerencekeli@hotmail.com; 5 Department of Pulmonary Medicine, Faculty of Medicine, Kocaeli University, Kocaeli 41001, Turkey; ilgazah@yahoo.com (A.I.); ersinalkilincmd@gmail.com (E.A.); ilknur.basyigit@gmail.com (I.B.); 6 Department of Pulmonary Medicine, Faculty of Medicine, Erciyes University, Kayseri 38038, Turkey; incigul@erciyes.edu.tr (I.G.); ebrutas29@hotmail.com (E.T.); 7 Department of Pulmonary Medicine, Konya Research and Training Hospital, Konya 42005, Turkey; burcu.samanyolu@yahoo.com (B.Y.); doktortaha@gmail.com (T.T.B.); 8 Department of Pulmonary Medicine, Dr. Suat Seren Chest Diseases and Thoracic Surgery Training and Research Hospital, Izmir 35170, Turkey; drglsn35@gmail.com (G.K.); nimetaksel@yahoo.com (N.A.); tatar.dursun@gmail.com (D.T.); 9 Department of Pulmonary Medicine, Atatürk Chest Diseases and Thoracic Surgery Training and Research Hospital, Ankara 06290, Turkey; yaseminkarabacakoglu@gmail.com (Y.S.); derya.ozaydin999@gmail.com (D.K.); 10 Department of Pulmonary Medicine, Faculty of Medicine, Dicle University, Diyarbakır 21280, Turkey; dr.haticesen@hotmail.com; 11 Department of Pulmonary Medicine, Faculty of Medicine, Van Yüzüncü Yıl University, Van 65080, Turkey; izciaysel@mynet.com (A.S.); buketmermit@gmail.com (B.M.); 12 Department of Pulmonary Medicine, Süreyyapaşa Chest Diseases and Thoracic Surgery Training and Research Hospital, İstanbul 34844, Turkey; sibel_boga@yahoo.com (S.B.); sibelarinc@yahoo.com.tr (S.A.); dildaryetis@yahoo.com (D.D.); muratkavas@gmail.com (M.K.); agcameltem@yahoo.com (M.A.); 13 Department of Pulmonary Medicine, Faculty of Medicine, Balıkesir University, Balıkesir 10145, Turkey; nurhangencer@hotmail.com; 14 Department of Pulmonary Medicine, Faculty of Medicine, Süleyman Demirel University, Isparta 32260, Turkey; ahbircan@yahoo.com (H.A.B.); hakanalp20@gmail.com (H.A.Y.); 15 Department of Pulmonary Medicine, Faculty of Medicine, Amasya University, Amasya 05100, Turkey; draylincapraz@yahoo.com; 16 Department of Pulmonary Medicine, Faculty of Medicine, Ufuk University, Ankara 06510, Turkey; aycnakbas@hotmail.com (A.Y.); esen_sayin@hotmail.com (E.S.G.); 17 Department of Pulmonary Medicine, Malatya Training and Research Hospital, Malatya 44000, Turkey; umutkasapoglu@gmail.com; 18 Department of Pulmonary Medicine, Necip Fazıl State Hospital, Kahramanmaraş 46050, Turkey; yararesra81@gmail.com (E.Y.); drfilizalkan@gmail.com (F.A.B.); 19 Department of Pulmonary Medicine, Kayseri State Hospital, Kayseri 38080, Turkey; alleynakemik@gmail.com (N.A.Y.); conteleno@gmail.com (C.B.); 20 Department of Pulmonary Medicine, Yedikule Chest Diseases and Thoracic Surgery Training and Research Hospital, İstanbul 34020, Turkey; fusunsahin19700@hotmail.com (F.S.); beratozturk@yahoo.com (B.U.); 21 Department of Pulmonary Medicine, Nigde Omer Halisdemir University Training and Research Hospital, Nigde 51100, Turkey; tabaruali@yahoo.com; 22 Department of Pulmonary Medicine, Bolvadin State Hospital, Bolvadin 03300, Turkey; gunhanyavasoglu@hotmail.com; 23 Department of Pulmonary Medicine, Faculty of Medicine, Uludağ University, Bursa 16059, Turkey; mehmetkarada@gmail.com (M.K.); gamzecelik9240@gmail.com (G.C.)

**Keywords:** chronic obstructive pulmonary disease, chronic bronchitis, pulmonary emphysema, disease exacerbation, phenotype

## Abstract

*Background and Objectives*: Chronic obstructive pulmonary disease (COPD) is heterogeneous, and phenotype-based classification may better capture differences in clinical burden and healthcare needs beyond standard GOLD categories. We aimed to describe the distribution of GesEPOC COPD phenotypes in Turkey and compare their demographic, clinical, functional, radiological, treatment, and healthcare utilization profiles. *Materials and Methods*: DIPTUR was a multicenter, observational, cross-sectional study conducted prospectively in 26 centers across 17 Turkish cities (October 2019–June 2021). Stable COPD patients (≥40 years; post-bronchodilator FEV1/FVC < 0.7) without exacerbation or major treatment modification within the previous four weeks were enrolled consecutively. Phenotypes were assigned per GesEPOC: exacerbator with emphysema (EE), exacerbator with chronic bronchitis (ECB), asthma–COPD overlap (ACO), and non-exacerbator (NE). Frequent exacerbators were defined as patients who experienced two or more exacerbations during the 12 months preceding enrollment, based on medical records and patient reports. *Results*: Among 894 patients, phenotype distribution was NE 44.1%, ECB 26.2%, EE 20.5%, and ACO 9.3%. Male predominance was observed across groups (80–89%; *p* = 0.006). Active smoking was most frequent in ECB (37.6%; *p* < 0.001), and BMI was lowest in EE (*p* < 0.001). Comorbidity patterns differed, with hypertension (*p* < 0.001), diabetes mellitus (*p* = 0.029), and heart failure (*p* < 0.001) most prevalent in ECB. Pulmonary function (FEV1 and FVC) was lowest in EE (both *p* < 0.001), and severe airflow limitation (GOLD III–IV) was most common in EE and ECB (*p* < 0.001). Dyspnea (mMRC ≥ 2) was more frequent in EE/ECB than in ACO/NE (*p* < 0.001). Emphysematous changes on thoracic CT predominated in EE (91.7%; *p* < 0.001). Long-term oxygen therapy was most common in EE (32.4%; *p* < 0.001). Emergency admissions, hospitalizations, and total length of stay were markedly higher in EE and ECB than in ACO and NE (all *p* < 0.001). *Conclusions*: COPD phenotypes in Turkey show substantial heterogeneity in clinical, functional, radiological, and utilization domains. Exacerbator phenotypes—particularly EE and ECB—represent higher-burden groups, supporting phenotype-oriented management and closer monitoring beyond GOLD classification.

## 1. Introduction

Chronic obstructive pulmonary disease (COPD) is common, heterogeneous, and globally burdensome, impairing health-related quality of life and causing substantial socioeconomic costs [1,2,3,4,5,6,7]. Beyond a single disease label, COPD manifests across distinct clinical phenotypes; recognizing these phenotypes is central to tailoring therapy and improving patient outcomes [1,3,8].

Current guidance emphasizes symptom burden and exacerbation history for staging disease severity [4,9]. Yet, the prognostic value of these categories for outcomes such as future exacerbations and mortality remains modest [10]. Consequently, phenotype-based classification has gained prominence as a pragmatic way to individualize care and refine risk stratification [4,11], with evidence that clinical phenotypes may better predict disease trajectory, guide treatment, and support prognostication than severity grades alone [6,12].

However, differentiating COPD phenotypes is challenging. Overlapping features complicate assignment [3], and nonuniform criteria produce divergent frequency estimates across studies and settings [3,4,9,10,13,14]. Critically, population-based data from Turkey remain limited, restricting external validity and local guideline adaptation [1].

Accordingly, the Distribution of COPD Phenotypes in Turkey (DIPTUR) study was designed to quantify the prevalence and distribution of COPD phenotypes in Turkey, examine environmental risk factors, and compare epidemiological, clinical, functional, and imaging characteristics across phenotypes. We also assessed the concordance between phenotype assignments and GOLD classifications, hypothesizing that COPD phenotypes in the Turkish population would exhibit distinct clinical, environmental, and functional profiles.

## 2. Methods

### 2.1. Study Design

**Figure 1:**
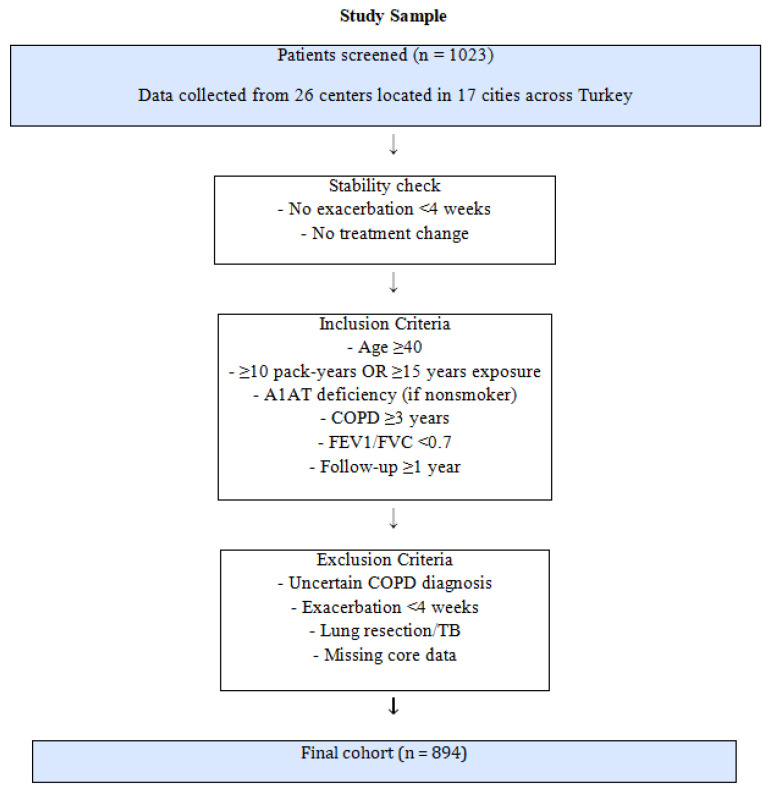
Flow chart of patient selection and formation of the study cohort.

This was a multicenter, observational, cross-sectional study conducted prospectively across 26 centers in 17 cities in Turkey between October 2019 and June 2021. The protocol was approved by the Karadeniz Technical University Faculty of Medicine Scientific Research Ethics Committee (16 September 2019, approval number 2019-244). The study adhered to the principles of the Declaration of Helsinki, and written informed consent was obtained from all participants. Consecutive eligible patients presenting during the study period were enrolled to minimize selection bias. A detailed list of participating centers, their geographic distribution, and patient contributions is provided in Figure 1.

### 2.2. Study Population and Eligibility Criteria

The study population consisted of consecutive patients with stable COPD who were under routine follow-up in outpatient clinics or inpatient wards at the participating centers during the study period. Stability was defined as the absence of an acute exacerbation or any major treatment modification within the four weeks preceding enrollment. Eligible patients were required to be at least 40 years of age, have a smoking history of ≥10 pack-years or at least 15 years of exposure to tobacco or biomass smoke, or, in the case of nonsmokers, a documented diagnosis of α-1 antitrypsin deficiency. Additional inclusion criteria included a physician-confirmed COPD diagnosis of at least three years’ duration, a post-bronchodilator FEV1/FVC ratio <0.7, and a documented clinical history of COPD with continuous clinical follow-up for at least one year prior to enrollment.

Patients were excluded if they had a suspected or uncertain diagnosis of COPD, an acute exacerbation within four weeks before enrollment, a history of lung resection or pulmonary tuberculosis, or incomplete core data, defined as key variables required for phenotype classification, including spirometry, clinical features, and exposure history. The inclusion and exclusion criteria were defined a priori to ensure a guideline-consistent, objectively confirmed COPD cohort and reliable phenotype assignment [15]: age ≥ 40 years and post-bronchodilator FEV1/FVC < 0.70 minimized diagnostic misclassification, enrollment in a stable state (no exacerbation or major treatment change within the prior 4 weeks) reduced acute event–related variability, and exclusion of prior lung resection or tuberculosis and incomplete data limited confounding and ensured standardized phenotyping.

In total, 1023 patients were screened, and after application of the eligibility criteria and exclusion of ineligible cases, the final analytic cohort comprised 894 patients (Figure 1).

### 2.3. COPD Phenotypes and Study Groups

COPD phenotypes were defined according to the Spanish COPD Guidelines (GesEPOC) [16], encompassing four subgroups: exacerbator with emphysema (EE), exacerbator with chronic bronchitis (ECB), asthma-COPD overlap features (ACO), and non-exacerbator (NE). Patients who experienced two or more exacerbations during the 12 months preceding enrollment were classified as frequent exacerbators [17]. The diagnosis of emphysema was based on thoracic computed tomography (CT) findings, including the presence of visually assessed emphysematous changes such as areas of low attenuation and parenchymal destruction, as reported in routine radiological evaluations. Chest X-ray findings alone were not used for emphysema classification.Phenotypic assignment was performed by experienced pulmonologists using a standardized clinical assessment form that incorporated clinical characteristics and thoracic CT findings. Prior to study initiation, all investigators underwent protocol training to ensure consistency in phenotype classification. Although formal inter-rater reliability testing was not performed, discrepancies were resolved by consensus. Based on these criteria, patients were categorized into four study groups corresponding to the defined phenotypes.

### 2.4. Data Collection Procedure

**Figure 2:**
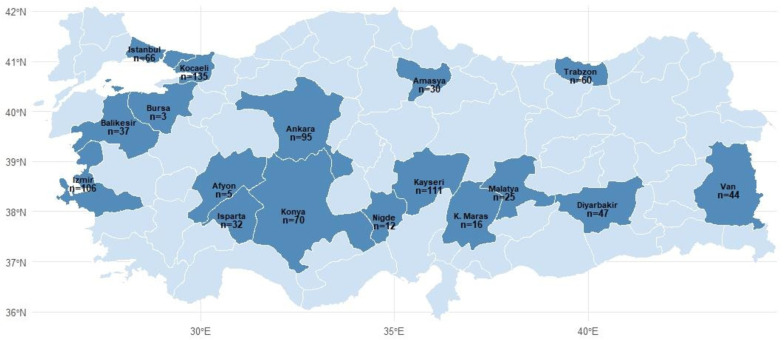
The representation of the centers included in the study.

All investigators received standardized protocol training at study initiation, and data entries were periodically audited to ensure uniformity across centers (Figure 2). Sociodemographic variables-including age, sex, marital status, education, body weight, height, and region of residence-were collected via face-to-face interviews or hospital records. Body mass index (BMI) was calculated as weight in kilograms divided by height in meters squared, and patients were classified as normal (<25.0 kg/m^2^), overweight (25.0–29.9 kg/m^2^), or obese (≥30.0 kg/m^2^) [18]. Smoking history was recorded, categorizing patients as nonsmokers, ex-smokers who quit ≥1 year, current smokers, or passive smokers. Environmental exposures, including biomass fuel use and occupational exposure to dust, gas, or smoke for over one year, along with family history of respiratory disease and comorbidities, were also documented [19].

Pulmonary function was assessed using the most recent post-bronchodilator spirometry within six months; if unavailable, testing followed American Thoracic Society/European Respiratory Society standards [20]. COPD severity was staged per GOLD 2021 criteria using percent predicted FEV1: stage 1 (≥80%), stage 2 (50–79%), stage 3 (30–49%), and stage 4 (<30%). Imaging reports, including chest radiographs and high-resolution CT scans within six months, were reviewed. Symptom burden and functional status were evaluated using the modified Medical Research Council (mMRC) dyspnea scale (scores ≥ 2 indicate significant dyspnea) [1,11] and the COPD Assessment Test (CAT) (scores ≥ 10 indicate clinically relevant symptoms) [1,11]. Maintenance and adjunctive pharmacological treatments during the stable phase were recorded. Health service utilization was captured by the number of emergency visits and hospitalizations due to COPD in the preceding year.

### 2.5. Statistical Analysis

All statistical analyses were performed using RStudio (version 2025.09.0). Descriptive statistics were expressed as median (minimum–maximum) for continuous variables and as frequency (n) and percentage (%) for categorical variables. The distribution of continuous variables was assessed using the Kolmogorov–Smirnov test. As the normality assumption was not satisfied for most variables, non-parametric statistical methods were applied. Comparisons of continuous variables across the four COPD phenotypes (EE, ECB, ACO, and NE) were performed using the Kruskal–Wallis test. When a statistically significant overall difference was identified, pairwise post hoc comparisons were conducted using the Mann–Whitney U test with Bonferroni correction, and statistically significant differences were denoted by superscript lettering. Categorical and ordinal variables were compared across phenotypic groups using chi-square test. All statistical tests were two-sided, and a *p*-value < 0.05 was considered statistically significant.

## 3. Results

A total of four COPD phenotypic subgroups were analyzed: exacerbator with emphysema (EE), exacerbator with chronic bronchitis (ECB), asthma–COPD overlap (ACO), and non-exacerbator (NE). Demographic and clinical characteristics of the study population are summarized in Table 1.

Median age differed significantly among phenotypes (*p* = 0.002), with higher values observed in the EE and ECB groups and lower values in the ACO and NE groups. A male predominance was present across all phenotypes, ranging from approximately 78% to 89%, with a significant difference in sex distribution between groups (*p* = 0.006).

Body mass index varied significantly across phenotypes (*p* < 0.001), being lowest in the EE group and higher in the ECB and ACO groups. Consistently, obesity was most prevalent in the ECB phenotype, while underweight or normal weight was more common in the EE group (*p* = 0.016). Regional distribution also differed significantly among phenotypes (*p* < 0.001), with patients in the EE and ECB groups more frequently originating from the Central Anatolian and Marmara regions.

Marital status differed significantly across phenotypes (*p* < 0.001), with married individuals constituting the majority in all groups and a higher proportion observed in the NE phenotype. Educational status varied across phenotypes (*p* = 0.001), with lower educational attainment more common in the EE group.

Smoking exposure differed significantly between groups (*p* < 0.001); active smoking was more frequent in the ECB phenotype, whereas non-smoking and passive exposure were relatively more common in the ACO group. In contrast, overall lifetime cigarette consumption, expressed as pack-years, did not differ significantly among phenotypes.

Comorbidities and environmental risk factors across COPD phenotypes are summarized in Table 2. A family history of COPD differed significantly among phenotypic groups (*p* = 0.002), with a higher frequency observed in the ACO and EE phenotypes and the lowest proportion in the NE group.

The prevalence of several comorbid conditions varied significantly across phenotypes. Hypertension was most frequent in the ECB group (50.2%) compared with the other phenotypes (*p* < 0.001). Similarly, diabetes mellitus showed significant differences between groups, with higher proportions observed in the ECB phenotype (*p* = 0.029). Heart failure also differed significantly across phenotypes (*p* < 0.001), being more common in the ECB group and least frequent in the ACO group. Arrhythmia showed a significant variation among groups (*p* = 0.002), with higher frequencies observed in the EE phenotype.

In contrast, the distribution of coronary artery disease, chronic liver disease, granulomatous diseases, chronic renal failure, solid organ malignancy, hematological malignancy, and chronic neurological disorders did not differ significantly among phenotypes (all *p* > 0.05).

Among coexisting respiratory conditions, bronchiectasis differed significantly across phenotypes (*p* < 0.001), with the highest prevalence observed in the ECB group. Obstructive sleep apnea did not show a significant difference between groups.

Occupational and environmental risk factors also varied across phenotypes. The proportion of patients with no known environmental risk differed significantly among groups (*p* = 0.030), whereas exposure to smoke and biomass and other environmental risks did not show significant differences.

Pulmonary function parameters and clinical assessment measures across COPD phenotypes are presented in Table 3. Significant differences were observed among phenotypic groups for all spirometric parameters, including FVC, FEV_1_, predicted FVC, predicted FEV_1_, and FEV_1_/FVC ratio (all *p* ≤ 0.003). Overall, lower absolute and predicted spirometric values were observed in the EE phenotype, whereas higher values were noted in the ECB, ACO, and NE phenotypes, with distinct intergroup differences indicated by post hoc comparisons.

The distribution of GOLD stages differed significantly across phenotypes (*p* < 0.001). More advanced airflow limitation (GOLD III–IV) was more frequently observed in the EE phenotype, whereas milder stages (GOLD I–II) were more common in the NE and ACO phenotypes.

Dyspnea severity assessed by the mMRC scale also showed significant variation among groups (*p* < 0.001). Higher mMRC grades were more prevalent in the EE and ECB phenotypes, while lower mMRC grades were more frequently observed in the ACO and NE phenotypes. Consistently, when mMRC scores were dichotomized, an mMRC score ≥2 was more common in the EE and ECB groups, whereas an mMRC score <2 was more frequently observed in the ACO and NE phenotypes (*p* < 0.001).

Thoracic imaging findings differed significantly across COPD phenotypes (Table 4). Emphysematous changes and normal CT findings differed significantly across phenotypes, with the highest frequencies observed in the EE group (*p* < 0.001). Acute pleuropulmonary disease was observed more frequently in the ECB phenotype (*p* = 0.008), while other imaging findings did not differ significantly among groups.

Treatment patterns varied significantly across phenotypes. Use of short- and long-acting bronchodilators, as well as combination inhaler therapies, differed among groups (all *p* < 0.05), whereas LAMA+ICS use showed no significant difference. Adjunctive medication use and oxygen supportive modalities differed significantly across phenotypes. BPAP and long-term oxygen therapy were more frequent in the EE and ECB groups (*p* < 0.001), with a modest but significant difference observed for CPAP use (*p* = 0.040).

Healthcare utilization and clinical outcomes differed significantly across COPD phenotypes (Table 5). The number of emergency admissions and hospitalizations due to COPD during the previous year was higher in the EE and ECB phenotypes compared with the ACO and NE phenotypes (both *p* < 0.001). Similarly, the length of hospital stay was longer in the EE and ECB groups, whereas shorter or no hospital stays were more frequently observed in the ACO and NE groups (*p* < 0.001).

The frequency of exacerbations also varied significantly among phenotypes (*p* < 0.001), with higher values observed in the EE and ECB phenotypes and lower values in the ACO and NE phenotypes.

## 4. Discussion

The findings of our study revealed significant heterogeneity among COPD phenotypes, underscoring the clinical importance of phenotypic classification in COPD. The COPD phenotypes without exacerbations were more common than those with exacerbations. Patients with the EE phenotype were characterized by advanced age, male gender, lower BMI, worse pulmonary function parameters, and a higher prevalence of stage IV COPD. Patients with COPD phenotypes with exacerbations, namely EE and ECB, had more severe symptom burden, poorer functional status evidenced by higher mMRC and CAT scores, and higher rates of emergency admissions and hospitalizations compared to patients with COPD phenotypes without exacerbations, namely ACO and NE. In contrast, patients with the NE phenotype exhibited milder disease, better pulmonary function, fewer comorbidities, and the lowest rates of emergency admissions and hospitalizations. The relatively higher DLCO% observed in the EE group should be interpreted with caution, as DLCO measurements were available only in a limited subset of patients; systematic testing in the entire cohort might have yielded lower DLCO% values in EE, consistent with emphysema pathophysiology. From a mechanistic perspective, the higher burden observed in exacerbator phenotypes-particularly EE and ECB-may reflect distinct pathological substrates, including parenchymal destruction with impaired gas exchange and hyperinflation in emphysema, and mucus hypersecretion with airway inflammation and infection susceptibility in chronic bronchitis, which together increase symptom severity and healthcare utilization. Clinically, these findings support phenotype-informed risk stratification to prioritize closer monitoring and proactive exacerbation prevention strategies in EE and ECB, beyond airflow limitation severity alone. These findings emphasize the need for phenotype-specific management strategies to improve clinical outcomes and optimize resource use in COPD care, in line with previous studies reporting that COPD phenotypes with frequent exacerbators, especially EE, were associated with more severe disease, highlighting the importance of targeted management approaches tailored to the predominant COPD phenotype.

As in our study, the most common and least common COPD phenotypes worldwide are reportedly the NE and ACO phenotypes, respectively, regardless of variations in COPD classification criteria used to categorize the phenotypes [1,5]. In a population-based study conducted in China, Bao et al. [4] reported that 90.3% of COPD patients had COPD phenotypes with exacerbations. Studies conducted in China and Latin America reported a lower prevalence of COPD phenotypes with acute exacerbations compared to those performed in European countries, as in our study [4,21,22]. The COPET study, a multicenter, cross-sectional, observational study conducted in Turkey, reported a comparable prevalence of COPD phenotypes [1]. However, unlike in our study, they noted that 17.7% of the patients had more than one phenotype, and 13.1% of the patients had no features of a specific phenotype. The discrepancies between studies in the prevalence of COPD phenotypes may be attributed to the COPD classification criteria used to categorize these phenotypes, as well as genetic and environmental risk factors.In addition, differences in healthcare access, diagnostic practices, smoking patterns, and environmental exposures may influence exacerbation recognition and reporting, thereby contributing to observed regional variability in phenotype distribution. Overall, the consistency of our findings with those of previously conducted national and international studies supports their generalizability to broader populations. Regional differences in environmental exposures and genetic predispositions may explain variations in the prevalence of COPD phenotypes.

The classification systems proposed for categorizing COPD phenotypes include multidimensional COPD phenotyping, GOLD-stage-specific COPD phenotyping, CT- and pulmonary function-based COPD phenotyping, or inflammatory parameters-based COPD phenotyping [10,13,19,23,24,25]. Several studies categorized COPD phenotypes based on the presence of exacerbations [26,27,28,29]. In comparison, we, as in several other studies, preferred the GesEPOC classification system due to its efficacy in distinguishing COPD phenotypes, given its integrated clinical and radiological criteria, and its practical applicability in clinical settings [1,4,5,6,7,8,12,21,30]. Our findings demonstrate that this integrated framework is clinically meaningful, as GesEPOC-defined phenotypes—particularly exacerbator phenotypes—were associated with clearly distinct clinical severity, pulmonary function impairment, symptom burden, and healthcare utilization patterns that were not fully explained by GOLD stage alone. From a mechanistic standpoint, the integration of exacerbation history with radiological features such as emphysema or chronic bronchitisappears to reflect divergent underlying disease substrates, allowingGesEPOC to capture distinct pathological pathways—parenchymal destruction versus airway-dominant disease—that are not fully reflected by airflow limitation or symptom scores alone. Clinically, this integrated approach was supported by our observation of substantially higher hospitalization rates, emergency admissions, and oxygen therapy use in EE and ECB phenotypes, facilitating more nuanced risk stratification and supports phenotype-oriented management decisions beyond GOLD stage categorization. However, given the challenges in diagnosing emphysema in population-based studies-such as the Phenotypes of COPD in Central and Eastern Europe (POPE) study-and considering the predominance of emphysema in patients with acute exacerbations without chronic bronchitis, defining this group as “acute exacerbators without chronic bronchitis (acute exacerbations with non-chronic bronchitis)” rather than “exacerbators with emphysema” may be a reasonable alternative [4,21,22]. Nevertheless, several authors have noted that a small proportion of patients with acute exacerbations cannot be clearly categorized as having either chronic bronchitis or emphysema [1]. Notably, such diagnostic overlap was not observed in our cohort, likely reflecting the availability of thoracic CT imaging and comprehensive clinical evaluation across centers, which strengthened phenotype assignment and internal consistency of the results.

Our categorization of COPD phenotypes was simple, clinically relevant, and easily applicable, highlighting the practicality of the GesEPOC classification system in clinical settings, particularly in tertiary care, where imaging and clinical details are more accessible compared to other systems that may face limitations in differentiating emphysema from chronic bronchitis in broader populations. From a clinical decision-making perspective, this practicality enables timely phenotype assignment at the point of care, supporting individualized treatment planning, risk stratification, and allocation of healthcare resources according to phenotype-specific disease burden.

We identified significant associations between COPD phenotypes and patients’ demographic, clinical, and laboratory characteristics. In line with several other studies [1,12,21,27,30], patients with COPD phenotypes featuring acute exacerbations exhibited a higher symptom burden, higher mMRC score, and increased rates of emergency admissions and hospitalizations, whereas patients without exacerbations demonstrated a milder disease course with better pulmonary function and lower healthcare utilization. These findings highlight distinct clinical profiles across COPD phenotypes and underscore the relevance of phenotypic classification for individualized disease management. Rather than merely reflecting disease severity, these phenotype-specific patterns may represent different trajectories of disease progression driven by underlying structural lung damage, airway inflammation, and systemic involvement, which together shape symptom burden and healthcare utilization. Due to the cross-sectional design of the study and the retrospective assessment of exacerbation history based on medical records and patient reports, causal relationships and temporal dynamics could not be established. Therefore, longitudinal studies are warranted to clarify causal pathways and to determine whether early phenotype-directed interventions can modify long-term outcomes. COPD phenotypes may also influence pharmacological treatment preferences. Medication usage patterns varied significantly across our study groups. The use of inhaled corticosteroids (ICS) was the highest in patients with COPD phenotypes featuring acute exacerbations, i.e., ECB and EE. Nonetheless, the optimal ICS indications in non-eosinophilic phenotypes remain debated. Higher use of short-acting muscarinic antagonist/short-acting β2-agonist combinations and triple therapy (long-acting muscarinic antagonist/long-acting β2-agonist + inhaled corticosteroids) in Groups EE and ECB than in Groups ACO and NE reflects the perceived need for aggressive symptom control and exacerbation prevention. From a clinical standpoint, this pattern likely mirrors physicians’ responses to higher exacerbation risk and symptom burden in EE and ECB, rather than a strictly phenotype-driven treatment algorithm. By contrast, long-acting β2-agonist/inhaled corticosteroid combinations use was the highest in Group ACO, in line with asthma-related treatment paradigms. The rate of patients not receiving any maintenance therapy was the highest in Group NE, likely reflecting their lower symptom burden and lower risk of exacerbation. However, Yazar et al. [1] reported that the type of COPD phenotype is not considered when determining the treatment modality. Taken together, our findings suggest a potential gap between real-world prescribing practices and phenotype-oriented management, indicating that systematic integration of phenotypic classification into treatment decision-making may improve therapeutic alignment and optimize exacerbation prevention strategies.

Key methodological strengths of our study include its multicenter design, encompassing a broad patient population nationwide, and its reliance on standardized COPD phenotypes based on clinical criteria, thereby enhancing the validity of our findings and enabling a reliable characterization of COPD phenotype distribution across Turkey. The inclusion of patients from diverse clinical settings further strengthens the external validity of the results and supports their applicability to routine clinical practice, particularly in healthcare systems where phenotype-based management is increasingly emphasized.

On the other hand, our study also had several limitations that should be acknowledged. First, its cross-sectional design did not allow assessing COPD phenotypes over time and establishing causal relationships between COPD phenotypes and the clinical, functional, and radiological findings. Longitudinal studies are needed to understand better the temporal dynamics and progression of each COPD phenotype. Accordingly, temporal dynamics and causal pathways could not be explored. Secondly, although we categorized COPD phenotypes based on the GesEPOC criteria, which are widely used in clinical practice, the absence of universally accepted definitions and the potential overlap between COPD phenotypes may have led to misclassification in some cases. Thirdly, our diagnoses of COPD phenotypes, namely emphysema and chronic bronchitis, were based solely on clinical and radiological criteria without quantitative imaging or biomarker-based measures, which might have further refined phenotype differentiation. Fourthly, although our study employed a multicenter design encompassing a large number of patients, the fact that it was conducted solely in Turkey may limit the generalizability of its findings to populations with different genetic backgrounds, environmental exposures, or healthcare practices. Fifthly, treatment modalities were recorded based on current prescriptions without assessing adherence, persistence, or longitudinal outcomes, which may have influenced the interpretation of phenotype-specific management patterns. Finally, the coronavirus-2019 (COVID-19) pandemic, which coincided with our study, led to substantial delays in data processing, analysis, and manuscript preparation.

## 5. Conclusions

In conclusion, the DIPTUR study confirms the heterogeneous nature of COPD by demonstrating that four distinct phenotypes (EE, ECB, ACO, and NE) exhibit significant differences in demographic, clinical, functional, and healthcare utilization characteristics. A key finding of this study is that frequent exacerbator phenotypes (EE and ECB) are characterized by more advanced age, lower lung function, higher comorbidity burden, and a disproportionate reliance on healthcare services, supporting their association with a more aggressive disease course. The novelty of this research lies in its systematic documentation of phenotype distribution within the Turkish population, providing specific insights into distinctive environmental exposure profiles and healthcare patterns. Furthermore, by highlighting the limited alignment between phenotype-based classification and the GOLD staging system, the study underscores that clinical phenotyping is a critical dimension for risk stratification beyond spirometric severity. These findings argue against a “one-size-fits-all” approach and provide a strong scientific rationale for adopting phenotype-driven, personalized treatment algorithms to optimize resource allocation and improve patient quality of life. Future research should focus on longitudinal cohorts to validate the stability of these phenotypes and develop evidence-based, phenotype-specific management guidelines through randomized controlled trials.

## Figures and Tables

**Table 1:** Demographic characteristics of patients across COPD phenotypes.

	Group				*p*
	EE	ECB	ACO	NE	
**Age** (year)	68 (44–93) ^a^	67 (45–100) ^ab^	65 (30–82) ^b^	65 (33–91) ^b^	**0.002**
**Sex**					
Male	161 (89)	185 (80.8)	65 (78.3)	328 (88.6)	**0.006**
Female	20 (11)	44 (19.2)	18 (21.7)	42 (11.4)	
**Body mass index** (kg/m^2^)	24.91 (14.88–43.76) ^a^	26.37 (15.35–51.14) ^b^	26.40 (15.20–40.79) ^b^	26.13 (14.13–61.83) ^b^	**<0.001**
**Obesity groups**					
Underweight/normal	91 (51.1)	86 (38.2)	29 (35.4)	148 (40)	**0.016**
Overweight	59 (33.1)	76 (33.8)	32 (39)	147 (39.7)	
Obese	28 (15.7)	63 (28)	21 (25.6)	75 (20.3)	
**Regional area**					
Marmara	42 (23.3)	57 (25)	19 (23.2)	110 (29.8)	**<0.001**
Aegean	28 (15.6)	23 (10.1)	14 (17.1)	51 (13.8)	
Mediterranean	3 (1.7)	14 (6.1)	4 (4.9)	8 (2.2)	
Central Anatolian	74 (41.1)	47 (20.6)	30 (36.6)	119 (32.2)	
Black Sea	12 (6.7)	22 (9.6)	11 (13.4)	53 (14.4)	
East Anatolian	20 (11.1)	34 (14.9)	2 (2.4)	16 (4.3)	
Southeast Anatolian	1 (0.6)	31 (13.6)	2 (2.4)	12 (3.3)	
**Marital status**					
Single	8 (4.4)	3 (1.3)	4 (4.8)	11 (3)	**<0.001**
Divorced/widowed	28 (15.5)	38 (16.6)	8 (9.6)	24 (6.5)	
Married	144 (79.6)	188 (82.1)	71 (85.5)	335 (90.5)	
**Educational status**					
Illiterate/primary	147 (81.2)	180 (78.6)	54 (65.1)	247 (66.8)	**0.001**
Secondary/college	31 (17.1)	44 (19.2)	23 (27.7)	106 (28.6)	
University or higher	3 (1.7)	5 (2.2)	6 (7.2)	17 (4.6)	
**Exposure to smoking**					
Active smoker	52 (28.7)	86 (37.6)	19 (22.9)	137 (37)	**<0.001**
Ex-smoker (>1 year)	115 (63.5)	106 (46.3)	44 (53)	198 (53.5)	
Non-smoker	8 (4.4)	22 (9.6)	10 (12)	23 (6.2)	
Passive exposure	6 (3.3)	15 (6.6)	10 (12)	12 (3.1)	
**History of cigarette** **consumption** (pack/year)	45 (8–150)	50 (10–130)	40 (15–70)	40 (5–120)	0.057
**History of previous** **cigarette consumption for ex-smokers** (pack/year)	40 (10–125) ^a^	40 (3–160) ^ab^	32.50 (2–120) ^b^	40 (3–150) ^ab^	**0.020**

Data presented as median (minimum–maximum) or *n* (%). Bold *p*-values indicate statistical significance. Different superscripts within the same row indicate statistically significant differences between phenotypic groups, whereas groups sharing the same superscript do not differ significantly. COPD: chronic obstructive pulmonary disease, EE: exacerbator with emphysema, ECB: exacerbator with chronic bronchitis, ACO: asthma-COPD overlap, NE: non-exacerbator.

**Table 2:** Comorbidities and environmental risk factors across COPD phenotypic subgroups.

	Group				*p*
	EE	ECB	ACO	NE	
**Family history of COPD**	46 (25.4)	47 (20.5)	26 (31.3)	53 (14.3)	**0.002**
**Coexisting diseases**					
Hypertension	65 (35.9)	115 (50.2)	29 (34.9)	124 (33.5)	**<0.001**
Diabetes mellitus	29 (16)	59 (25.8)	15 (18.1)	58 (15.7)	**0.029**
Heart failure	21 (11.6)	49 (21.4)	4 (4.8)	25 (6.8)	**<0.001**
Coronary artery disease	42 (23.2)	49 (21.4)	13 (15.7)	56 (15.1)	0.068
Chronic liver disease	1 (0.6)	5 (2.2)	0 (0)	10 (2.7)	0.168
Arrhythmia	18 (9.9)	11 (4.8)	2 (2.4)	8 (2.2)	**0.002**
Granulomatous diseases	14 (7.7)	12 (5.2)	6 (7.2)	13 (3.5)	0.207
Chronic renal failure	8 (4.4)	8 (3.5)	5 (6)	13 (3.5)	0.765
Solid organ cancer (past/present)	12 (6.6)	20 (8.7)	5 (6)	38 (10.3)	0.309
Hematological malignancy (Past/present)	3 (1.7)	3 (1.3)	1 (1.2)	3 (0.8)	0.686
Chronic neurological disorders	4 (2.2)	3 (1.3)	1 (1.2)	4 (1.1)	0.504
**Coexisting with other diseases**					
Obstructive sleep apnea	3 (1.7)	10 (4.4)	0 (0)	7 (1.9)	0.171
Bronchiectasis	27 (14.9)	67 (29.3)	14 (16.9)	54 (14.6)	**<0.001**
**Occupational/environmental risk factors**					
No known risks	59 (32.6)	94 (41)	31 (37.3)	114 (30.8)	**0.030**
Exposure to smoke and biomass	36 (19.9)	53 (23.1)	22 (26.5)	55 (14.9)	0.085
Other risks	8 (4.4)	7 (3.1)	5 (6)	20 (5.4)	0.525

Data presented as *n* (%). Bold *p*-values indicate statistical significance. COPD: chronic obstructive pulmonary disease, EE: exacerbator with emphysema, ECB: exacerbator with chronic bronchitis, ACO: asthma-COPD overlap, NE: non-exacerbator.

**Table 3:** Pulmonary function parameters and clinical assessment measures across COPD phenotypic subgroups.

	Group				*p*
	EE	ECB	ACO	NE	
**Forced vital capacity (FVC) (L)**	2.22 (0.72–4.31) ^a^	2.38 (0.56–5.50) ^b^	2.38 (1.14–4.51) ^bc^	2.38 (0.53–6.61) ^cd^	**<0.001**
**Predicted FVC (%)**	62 (0.33–114) ^a^	64.68 (0.42–182) ^a^	64.68 (9–131) ^b^	64.68 (19–142) ^b^	**<0.001**
**Forced expiratory volume in 1 sec (FEV1) (L)**	1.13 (0.32–2.76) ^a^	1.34 (0.32–3.10) ^b^	1.34 (0.52–3.34) ^bc^	1.34 (0.40–3.72) ^c^	**<0.001**
**Predicted FEV1 (%)**	43 (12–91) ^a^	48.88 (17–94) ^b^	48.88 (19–96) ^bc^	48.88 (9–110) ^c^	**<0.001**
**FEV1/FVC (%)**	60 (27.60–70) ^a^	60 (29–70) ^ab^	60 (29–70) ^ab^	60 (29–70) ^b^	**0.003**
**Groups of the GOLD stage**					
I (predicted FEV1 ≥ 80%)	4 (2.8)	7 (4.7)	5 (7.6)	24 (7.8)	**<0.001**
II (predicted FEV1 ≥ 50%, <80%)	28 (19.6)	53 (35.3)	28 (42.4)	153 (50)	
III (predicted FEV1 ≥ 30%, <50%)	63 (44.1)	61 (40.7)	22 (33.3)	103 (33.7)	
IV (predicted FEV1 < 30%)	48 (33.6)	29 (19.3)	11 (16.7)	26 (8.5)	
**Groups of the mMRC stage**					
0	4 (2.2)	7 (3.1)	7 (8.4)	39 (10.5)	**<0.001**
1	27 (14.9)	30 (13.2)	22 (26.5)	147 (39.7)	
2	54 (29.8)	56 (24.7)	26 (31.3)	103 (27.8)	
3	68 (37.6)	78 (34.4)	20 (24.1)	59 (15.9)	
4	28 (15.5)	56 (24.7)	8 (9.6)	22 (5.9)	
**mMRC groups**					
<2	31 (17.1)	37 (16.3)	29 (34.9)	186 (50.3)	**<0.001**
≥2	150 (82.9)	190 (83.7)	54 (65.1)	184 (49.7)	

Data presented as median (minimum–maximum) or *n* (%). Bold *p*-values indicate statistical significance. Different superscripts within the same row indicate statistically significant differences between phenotypic groups, whereas groups sharing the same superscript do not differ significantly. EE: exacerbator with emphysema, ECB: exacerbator with chronic bronchitis, ACO: asthma-COPD overlap, NE: non-exacerbator, mMRC: Modified Medical Research Council Dyspnea Scale.

**Table 4:** Thoracic imaging findings and therapeutic management across COPD phenotypic subgroups.

	Group				*p*
	EE	ECB	ACO	NE	
Computed tomography of the thorax					
Normal	151 (83.4)	174 (76)	61 (73.5)	265 (71.6)	**0.025**
Amphisematous changes	166 (91.7)	96 (41.9)	34 (41)	215 (58.1)	**<0.001**
Inactive tuberculosis	24 (13.3)	28 (12.2)	8 (9.6)	39 (10.5)	0.732
Interstitial lung disease	3 (1.7)	8 (3.5)	2 (2.4)	4 (1.1)	0.190
Pleural thickening and calcific plaque	14 (7.7)	23 (10)	6 (7.2)	18 (4.9)	0.114
Atelectasia	7 (3.9)	21 (9.2)	7 (8.4)	39 (10.5)	0.070
Solid or cystic lesions	14 (7.7)	11 (4.8)	4 (4.8)	23 (6.2)	0.620
Acute pleuropulmonary disease	10 (5.5)	26 (11.4)	5 (6)	16 (4.3)	**0.008**
Bronchiectasis	25 (13.8)	47 (20.5)	13 (15.7)	50 (13.5)	0.118
Others	20 (11)	23 (10)	9 (10.8)	29 (7.8)	0.582
**Inhaled Medication Regimens**					
No medication	171 (95.5)	212 (93)	77 (92.8)	320 (87)	**0.004**
SABA/SAMA, SABA+SAMA	119 (66.5)	122 (53.5)	22 (26.5)	70 (19)	**<0.001**
LABA	60 (33.5)	70 (30.7)	10 (12)	25 (6.8)	**<0.001**
LAMA	74 (41.3)	63 (27.6)	14 (16.9)	79 (21.5)	**<0.001**
LABA+LAMA	7 (3.9)	27 (11.8)	9 (10.8)	56 (15.2)	**0.002**
LABA+ICS	90 (50.3)	70 (30.7)	15 (18.1)	80 (21.7)	**<0.001**
LAMA+ICS	1 (0.6)	2 (0.9)	3 (3.6)	9 (2.4)	0.146
LABA+LAMA+ICS	65 (36.3)	79 (34.6)	32 (38.6)	95 (25.8)	**0.016**
**Adjunctive medications**					
Oral corticosteroids	4 (2.2)	0 (0)	3 (3.6)	3 (0.8)	**0.015**
Others (theophylline, roflumilast)	23 (12.8)	24 (10.5)	14 (16.9)	19 (5.2)	**0.001**
**Oxygen supportive modalities**					
CPAP	2 (1.1)	4 (1.8)	0 (0)	0 (0)	**0.040**
BPAP	20 (11.2)	19 (8.3)	1 (1.2)	1 (0.3)	**<0.001**
Long-term oxygen therapy	58 (32.4)	58 (25.4)	3 (3.6)	8 (2.2)	**<0.001**

Data presented as *n* (%). Bold *p*-values indicate statistical significance.EE: exacerbator with emphysema, ECB: exacerbator with chronic bronchitis, ACO: asthma-COPD overlap, NE: non-exacerbator, SABA: short-acting β2-agonists, SAMA: short-acting muscarinic antagonist; LABA: long-acting β2-agonists, LAMA: long-acting muscarinic agonists, ICS: inhaled corticosteroids, CPAP: continuous positive airway pressure, BPAP: bilevel positive airway pressure.

**Table 5:** Healthcare utilization and clinical outcomes across COPD phenotypic subgroups.

	Groups				*p*
	EE	ECB	ACO	NE	
Emergency admission due to COPD during the last year					
Number of admissions	2 (0–30) ^a^	2 (0–35) ^a^	0 (0–20) ^b^	0 (0–1) ^c^	**<0.001**
**Hospitalization due to COPD during the last year**					
Number of hospitalizations	1 (0–15) ^a^	1 (0–30) ^a^	0 (0–5) ^b^	0 (0–3) ^c^	**<0.001**
Length of hospital stays	10 (0–150) ^a^	7 (0–143) ^a^	0 (0–80) ^b^	0 (0–23) ^c^	**<0.001**
Frequent exacerbations	2 (2–26) ^a^	2 (2–16) ^a^	0 (0–0.20) ^b^	0 (0–1) ^c^	**<0.001**

Data presented as median (minimum–maximum). Bold *p*-values indicate statistical significance. Different superscripts within the same row indicate statistically significant differences between phenotypic groups, whereas groups sharing the same superscript do not differ significantly. EE: exacerbator with emphysema, ECB: exacerbator with chronic bronchitis, ACO: asthma-COPD overlap, NE: non-exacerbator, COPD: chronic obstructive pulmonary disease.

## Data Availability

The datasets generated and analyzed during the current study are not publicly available due to patient privacy regulations but are available from the corresponding author upon reasonable request and with appropriate ethical approvals.
